# Granulomatous Thyroiditis Post Tuberculosis Infection Mimicking a Neoplasm

**DOI:** 10.7759/cureus.69847

**Published:** 2024-09-21

**Authors:** Swathi Srinivas, Nagarajan Priyathersini, Archana Balasubramanian, Rajiv Raj D

**Affiliations:** 1 College of Medicine, Sri Ramachandra Institute of Higher Education and Research, Chennai, IND; 2 Pathology, Sri Ramachandra Medical College and Research Institute, Chennai, IND; 3 Pathology, Sri Ramachandra Institute of Higher Education and Research, Chennai, IND; 4 General Surgery, Sri Ramachandra Institute of Higher Education and Research, Chennai, IND

**Keywords:** fine-needle aspiration, granuloma, inflammation, neoplasm, thyroid, tuberculosis

## Abstract

Granulomatous thyroiditis is a diffuse inflammation of the thyroid gland that has a wide range of etiologies like viral, bacterial, and autoimmune conditions. The most common type is subacute granulomatous thyroiditis due to viral infection, seen in the middle-aged group and affects women. A 65-year-old woman, presented with complaints of anterior neck swelling for three months. Due to lack of pain, her age, a positive family history, and atypical cells on workup, she was posted for total thyroidectomy and the specimen was sent for histopathological examination (HPE). The report showed nodular goiter with focal granulomas, and a diagnosis of granulomatous thyroiditis was made. Post-operative CT chest showed scarring of the lung, so the etiology was narrowed down to tuberculosis. Granulomatous thyroiditis mimicking a neoplasm is uncommon in the elderly. A detailed workup must be done to identify the etiology and treat any underlying infection.

## Introduction

Thyroiditis includes several clinical disorders characterized by inflammation of the thyroid gland [1]. It can be associated with normal, elevated, or depressed thyroid function [2]. Thyroiditis can manifest as a painful or painless swelling. The most common etiologies of painful thyroiditis include infection, radiation, or trauma, and painless thyroiditis includes auto immune conditions and iatrogenic or idiopathic fibrosis [2].

The most common granulomatous disease of the thyroid is subacute granulomatous thyroiditis, which is due to viral or post-viral inflammation [3]. It was first described by Dr. Fritz de Quervain in 1902 [4].

Granulomatous thyroiditis commonly presents with diffuse neck pain. Initially, there will be a phase of hyperthyroidism attributed to the destruction of follicles and release of colloid followed by a hypothyroid state due to a decrease of thyroid-stimulating hormone from the pituitary gland [3].

Diagnosis is made through a detailed history taking and physical examination. The presence or absence of pain is an important history for differentials and must be elicited. Investigations include thyroid function tests, tests for antibodies, and other routine investigations. Fine-needle aspiration is done in cases of painful swelling or if there is suspicion of malignancy [3].

In granulomatous thyroiditis, cytologic examination typically shows clusters of epithelioid histiocytes (granulomas) with a predominance of neutrophils in the background [5,6].

In the presence of pain, management is primarily focused on reducing the pain and restoration of euthyroidism [2]. In suspected cases of malignancy, a thyroidectomy can be done, and the sample must be subjected to histopathology to decide the further course of treatment. in cases of acute airway obstruction and wheezing, an isthmectomy can be performed in order to relieve the pressure in the trachea.

Tuberculosis of the thyroid gland is rare. Tuberculosis causes granulomatous thyroiditis, but it is different compared to subacute DeQuervain thyroiditis, which is also granulomatous but due to a viral infection. The diagnosis is very difficult because the clinical presentation has no clear-cut characteristics. The clinical course may resemble toxic goiter or acute thyroiditis or may follow a subacute pattern with no defined symptoms [7]. Staining is usually negative in tissue specimens in various conditions like increased age, decreased CD8+ count, and absence of extrapulmonary tuberculosis [8].

Here, we present the case of an elderly woman, who presented with a swelling on the anterior aspect of the neck, which was initially suspected to be malignancy but was finally reported as subacute granulomatous thyroiditis after tuberculosis.

## Case presentation

A 65-year-old female presented to our medical center with swelling over the front of the neck for three months. The swelling was not associated with pain. The patient did not give any history of fever, weight loss, cough, dyspnea, or hemoptysis. She did not have any wheezing, signs of airway obstruction, or deviation of the trachea. Family history was significant for endocrine malignancies, particularly thyroid malignancy. On examination, a swelling of size 6 cm x 5 cm was palpable in the anterior aspect of the left side of the neck and moved with deglutition. The patient was subjected to thyroid function tests and other routine investigations, which came back to be normal. Ultrasonography of the neck revealed an enlarged left lobe of the thyroid with a large adenoma and no lymph node involvement. Fine-needle aspiration of the left thyroid nodule showed granulomas and atypia of undetermined significance (Bethesda category III). Differential diagnoses were narrowed down to follicular adenoma of the thyroid gland or carcinoma thyroid (in view of family history).

The patient successfully underwent a total thyroidectomy and the gross examination of the left lobe was enlarged measuring 11 cm x 7 cm x 4 cm. The sample along with the attached isthmus (M) 4 cm x 2.5 cm x 1 cm was submitted for histopathological examination.

Gross appearance showed the external surface of the left lobe and part of the isthmus appear gray, brown, and smooth. The cut surface of the left lobe appeared gray and brown. Multiple gray, white firm areas were noted. Focal gray and black areas were seen. The cut surface of the isthmus was gray, brown, and unremarkable.

**Figure 1:**
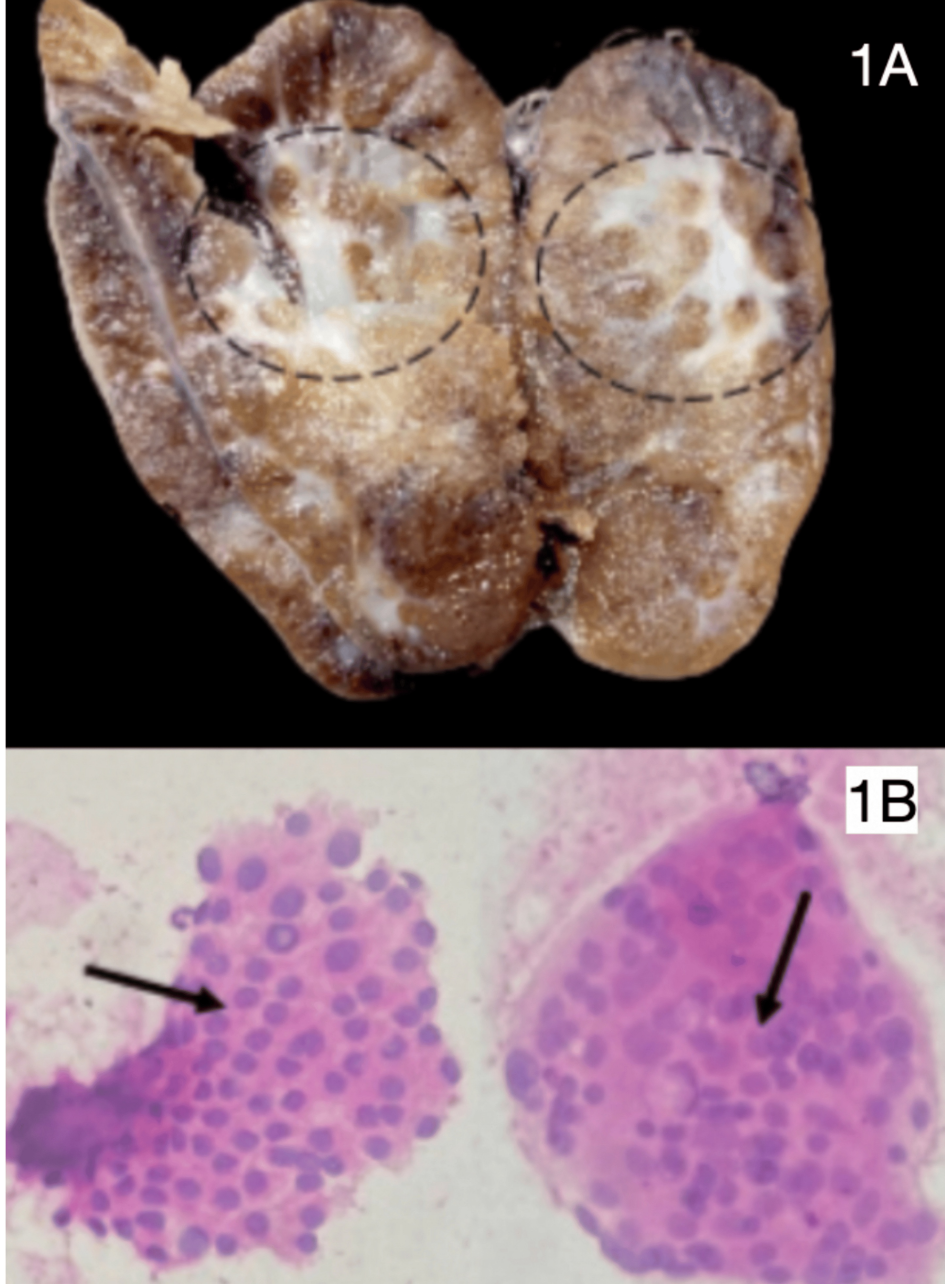
Gross and cytology images of the thyroid gland (hematoxylin and eosin staining used) 1A: A cut section of the left lobe of the thyroid showing gray-white sclerotic areas. 1B: Cytology smears show pseudo inclusions (left) and nuclear overlapping with grooving (right) (10x magnification).

As in Figure 1, the gross appearance of the right lobe (M) is 4.5 cm x 2.5 cm x 1.5 cm with the attached isthmus (M) of 1.8 cm x 1 cm x 0.4 cm. The external surfaces of both are gray, brown, and smooth. The cut surface of the right lobe is gray and brown. Two cysts were noted, one measuring 1.4 cm x 1.2 cm and the other measuring 0.8 cm x 0.5 cm. The rest of the parenchyma was noted to be gray, brown, and unremarkable.

**Figure 2:**
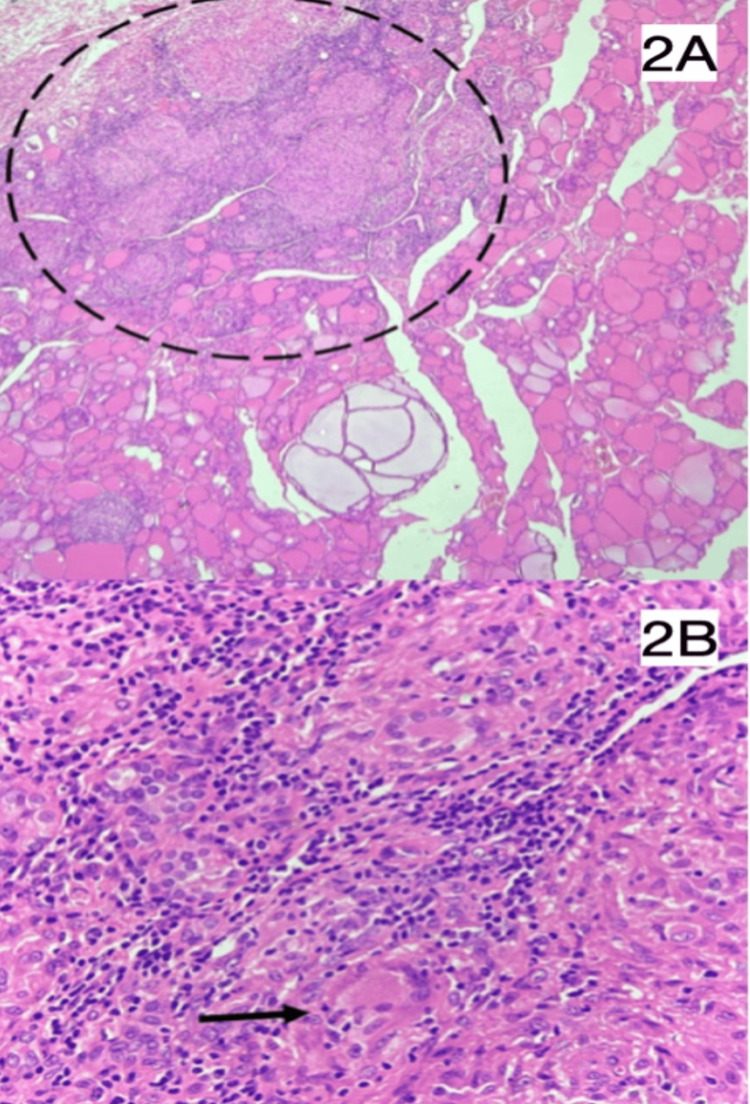
Histopathology images of the thyroid gland (hematoxylin and eosin staining used) 2A: Low-power (10x magnification) image showing thyroid follicles with adjacent granuloma. 2B: High-power (40x magnification) image of granulomatous inflammation with giant cells, epithelioid cells, and lymphocytes.

As shown in Figure 2, the microscopy showed thyroid parenchyma with follicles of varying sizes filled with colloids, while the surrounding areas had extensive sclerosis. Section from the isthmus revealed multiple epithelioid granulomas with multi-nucleated giant cells and peripheral lymphocyte cuffing. Furthermore, one lymph node showed reactive changes, while there was no evidence suggestive of malignancy. This led to the differentials being narrowed down to De Quervain’s thyroiditis and tuberculosis-associated inflammation.

Special stains for acid-fast bacteria were negative. During post-operative management, the computed tomography chest revealed an old scarring of lung parenchyma, and the patient was empirically started on anti-tubercular therapy.

## Discussion

We present a case of granulomatous thyroiditis after tuberculosis. It was initially suspected to be malignancy due to her age, a positive family history, and atypical cells found in fine-needle aspiration. In view of these factors, she was subjected to total thyroidectomy. Further histopathological evaluation of the specimen revealed granulomas and predominant neutrophil infiltration and a diagnosis of subacute granulomatous thyroiditis was made. AFB staining of the tissue was negative, but on CT chest, old scarring of lung parenchyma was spotted, and the patient was empirically started on anti-tuberculosis treatment (ATT).

Subacute granulomatous thyroiditis (also known as giant cell thyroiditis, subacute thyroiditis, or De Quervain’s thyroiditis) is the most common cause of thyroid pain [9]. More commonly, it affects women as much as four times more than men and commonly occurs at 40 to 50 years of age [9]. The most common etiology is a viral infection. The summer peak incidence of thyroiditis usually coincides with the peak incidences of coxsackievirus groups A and B and echovirus infections [10]. We attribute this patient’s symptoms of thyroiditis to either an undiagnosed tuberculosis infection or a latent viral infection.

The symptoms of subacute granulomatous thyroiditis include a prodrome of myalgia, pharyngitis, low-grade fever, and fatigue, followed by a tender, diffusely enlarged swelling in the neck associated with pain. The classical course is hyperthyroidism followed by hypothyroidism.

Thyroid-stimulating hormone (TSH) should be checked to check the thyroid status of the patient. Thyroid microsomal antibodies and thyroid receptor antibodies can be checked to rule out autoimmune thyroid disease. Erythrocyte sedimentation rate (ESR) and C-reactive protein (CRP) are significantly elevated in infectious thyroiditis [9].

Thyroid ultrasound is the most important imaging modality for the evaluation of the thyroid gland. Heterogenous thyroid parenchyma indicates inflammation of the thyroid gland, but it does not differentiate between production versus destruction of thyroiditis. It can also detect nodules in the thyroid gland [10]. A thyroid uptake scan can be done to differentiate production thyroiditis or destruction thyroiditis in patients who present with thyrotoxicosis characterized by low TSH with or without elevated T4 and T3. Low uptake on thyroid scan would indicate destruction thyroiditis, while increased or normal uptake indicates production thyroiditis.

Fine-needle aspiration is done to evaluate suspicious thyroid nodules to rule out malignancy. In general, any thyroid nodule, which is less than 10 mm, can be monitored without fine-needle aspiration [6].

Treatment primarily consists of relieving thyroid pain and tenderness with nonsteroidal anti-inflammatory drugs (NSAIDs). Secondary treatment involves identifying the etiology and treating any unresolved infection. The average duration of treatment is around five weeks [9].

## Conclusions

Subacute granulomatous thyroiditis is a common cause of anterior neck swelling. It occurs during or after a viral infection. The most common trajectory of the disease is hyperthyroidism followed by hypothyroidism. Diagnostic workup includes routine investigations, ESR, CRP, fine-needle aspiration, thyroid function test, and test for the presence of antibodies. Other types of aggressive thyroid malignancies such as anaplastic carcinoma or thyroid angiosarcoma have to be ruled out. Management includes symptomatic treatment for the pain and hyperthyroidism. Euthyroidism is expected to return in six to 12 months. Thus, granulomatous thyroiditis can be attributed to subacute DeQuervain thyroiditis but also, in rare cases, to an extrapulmonary manifestation of tuberculosis. Clinicians should always be aware of tuberculosis in cases of granulomatous thyroiditis.
